# Prognostic significance of KN motif and ankyrin repeat domains 1 (KANK1) in invasive breast cancer

**DOI:** 10.1007/s10549-019-05466-8

**Published:** 2019-11-02

**Authors:** Yousif A. Kariri, Chitra Joseph, Sasagu Kurozumi, Michael S. Toss, Mansour Alsaleem, Sara Raafat, Nigel P. Mongan, Mohammed A. Aleskandarany, Andrew R. Green, Emad A. Rakha

**Affiliations:** 1 Division of Cancer and Stem Cells, School of Medicine, Nottingham City Hospital, The University of Nottingham, Nottingham, UK; 2 Faculty of Applied Medical Science, Shaqra University, Riyadh, Saudi Arabia; 3 Cancer Biology and Translational Research, Faculty of Medicine and Health Sciences, University of Nottingham, Nottingham, UK; 4 Department of Pharmacology, Weill Cornell Medicine, New York, USA; 5 Department of Histopathology, Nottingham University Hospital NHS Trust, City Hospital Campus, Hucknall Road, Nottingham, NG5 1PB UK

**Keywords:** Invasive breast cancer, Lymphovascular invasion, KANK1, Prognostic

## Abstract

**Background:**

KN motif and ankyrin repeat domains 1 (KANK1) plays an important role in cytoskeleton maintenance and contributes to the regulation of cell proliferation, adhesion and apoptosis. KANK1 is involved in progression of a variety of solid tumours; however, its role in invasive breast cancer (BC) remains unknown. This study aims to evaluate the clinicopathological and prognostic value of KANK1 expression in operable BC.

**Methods:**

KANK1 expression was assessed at the transcriptomic level using multiple BC cohorts; the Molecular Taxonomy of BC International Consortium cohort (METABRIC; *n* = 1980), The Cancer Genome Atlas BC cohort (TCGA; *n* = 949) and the publicly available BC transcriptomic data hosted by BC Gene-Expression Miner (bc-GenExMiner v4.0) and Kaplan–Meier plotter?. The Nottingham BC cohort (*n* = 1500) prepared as tissue microarrays was used to assess KANK1 protein expression using immunohistochemistry (IHC). The association between clinicopathological variables and patient outcome was investigated.

**Results:**

In the METABRIC cohort, high expression of *KANK1* mRNA was associated with characteristics of good prognosis including lower grade, absence of lymphovascular invasion and HER2 negativity (all; *p* < 0.001) and with better outcome [*p* = 0.006, Hazards ratio, (HR) 0.70, 95% CI 0.54–0.91]. High KANK1 protein expression was correlated with smaller tumour size and HER2 negativity, and better outcome in terms of longer breast cancer-specific survival [*p* = 0.013, HR 0.7, 95% CI 0.536–0.893] and time to distant metastasis [*p* = 0.033, HR 0.65, 95% CI 0.51–0.819].

**Conclusion:**

These results supported that upregulation of KANK1 works as a tumour suppressor gene in BC and is associated with improved patients’ outcomes.

**Electronic supplementary material:**

The online version of this article (10.1007/s10549-019-05466-8) contains supplementary material, which is available to authorized users.

## Background

Breast cancer (BC) is a heterogeneous disease associated with a variety of morphological, molecular features, outcomes and response to therapy [1]. Although BC outcome has improved over the years, 20–30% of patients develop distant metastasis with subsequent poor outcome [2]. Several mechanisms are involved in BC metastasis; however, the key molecular factors driving metastasis remain to be defined.

KN motif and ankyrin repeat domains 1 gene (*KANK1*) is located at chromosome 9p24 [3] and is composed of KANK N-terminal (KN) motif, the central coiled-coil domains and the C-terminal ankyrin (ANK) repeats [4]. Notably, KANK1 protein interacts with other associated proteins via the coiled-coil and the ankyrin repeat domains, respectively [4]. KANK1 has an essential role in cytoskeleton maintenance via regulating the rate of cytoskeleton proteins production and controlling actin polymerisation [4]. KANK1 plays an important role in the down-regulation of the Rho-associated kinase (ROCK) pathway [5], which is recognised to be involved in various cellular functions such as proliferation, adhesion, cell differentiations and apoptosis [6]. This allow KANK1 to integrate alongside with β-catenin aiming to regulate its distribution in the nucleus and concentrate its transcription, therefore, affecting the development of cancer [7]. Importantly, several in vivo studies revealed a link between the ROCK pathway and tumour cell metastasis [8, 9] and indicated its role in multiple human cancers including BC [6].

The signalling processes controlled by KANK1 expression are also involved in the regulation of epithelial mesenchymal transmission (EMT) by cooperating with transforming growth factor-β (TGF-β) to induce the cytoskeletal reorganisation [10]. KANK1 plays an important role in the development of many malignant tumours. For instance, in vivo KANK1 overexpression reduces the tumorigenicity in lung cancer [11]. Further, in vivo and in vitro studies confirm that KANK1 upregulation in gastric cancer leads to a decrease in the metastatic ability of tumour cells [12]. However, the prognostic significance of KANK1 expression in BC remains unclear. This study aimed to assess the biological and clinical significance of *KANK1* mRNA and KANK1 protein expression in BC and the association between *KANK1* mRNA expressions with EMT-related genes.

## Materials and methods

### Study cohorts

#### *KANK1* transcriptomic data

The molecular taxonomy of breast cancer international consortium (METABRIC) dataset (*n* = 1980) [13] was used to evaluate *KANK1* mRNA expression. In the METABRIC, mRNA extracted from primary tumour samples was assayed using the Illumina Human HT-12 v3 platforms (Illumina, Inc., San Diego, USA). Gene-expression data were prepared and normalised as described previously [14]. Furthermore, The Cancer Genome Atlas (TCGA) BC dataset (*n* = 895) [15] was used to evaluate *KANK1* mRNA expression. In the TCGA cohort, RNASeqV2 data and clinicopathological information provided by cBioPortal were used [16, 17]. The prognostic value of *KANK1* mRNA expression was further evaluated using the online Breast Cancer Gene-Expression Miner v4.0 (bc-GenExMiner v4.0) database (*n* = 3871) [18] and the Kaplan–Meier plotter (*n* = 1402) [19].

### KANK1 protein expression

#### KANK1 protein cohort

A well-characterised cohort of primary operable BC was incorporated in this study, in which the cases were collected from patients presented to Nottingham City Hospital, NHS Trust between 1998 and 2006 (Supplementary Table 1). The Nottingham Prognostic Index (NPI) and oestrogen receptor (ER) status were used to classify patients into clinically relevant groups for management purposes. Based on the NPI, patients were sub-classified into two groups; patients with NPI > 3.4 received tamoxifen if ER status was positive and chemotherapy if ER was negative; however, patients who had NPI ≤ 3.4 received no adjuvant therapy. Patients lacking ER expression (ER) and eligible to receive chemotherapy were treated with classical cyclophosphamide, methotrexate and 5-flurouacil (CMF). Neoadjuvant therapy or anti-Her2-targeted therapy was not used to treat patients in this study. Information of therapy, clinical history and outcomes are prospectively maintained. Outcome data included development and time to distant metastasis (TTDM) and breast cancer-specific survival (BCSS) [20]. BCSS was defined as the duration (in months) from the date of primary surgery to the time of death because of BC. Distant metastasis-free interval was defined as the duration (in months) from primary surgical treatment to the occurrence of first distant recurrence. The distribution of clinicopathological parameters between the discovery cohort (METABRIC) and the validation cohort (Nottingham) presented no statistical differences (all correlation coefficients (*r*) = 0.80, all *p* < 0.001).

### Immunohistochemistry (IHC)

**Fig. 1:**
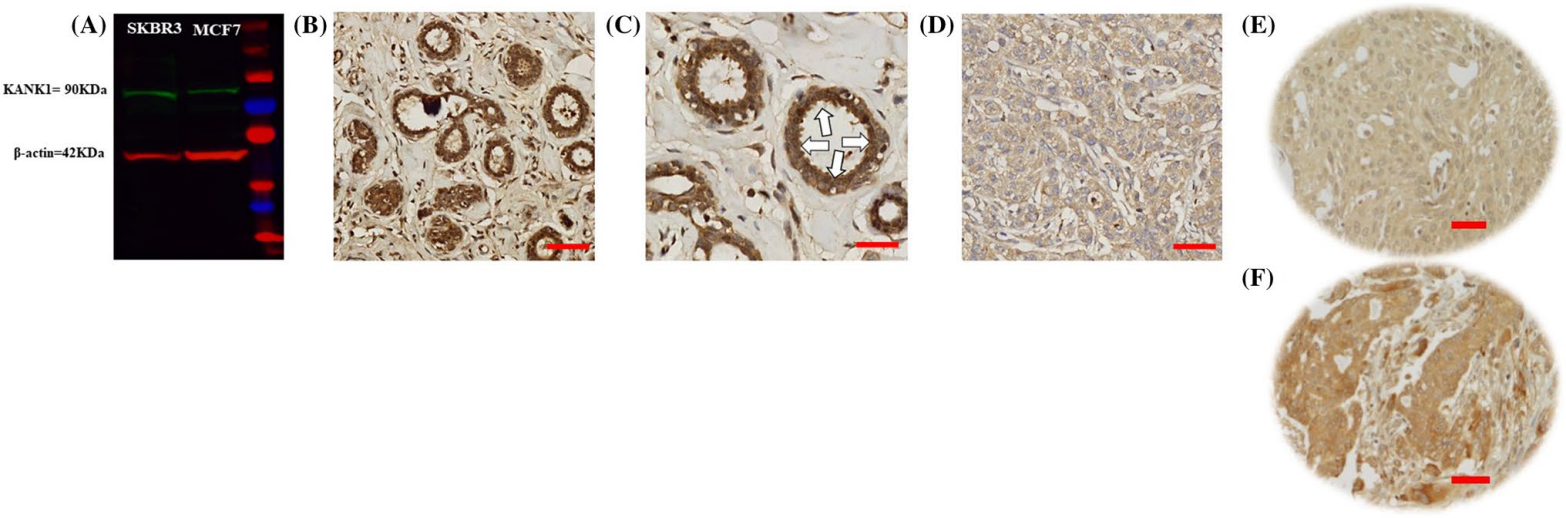
Western blot and immunohistochemical expression of KANK1 in BC. **a** Western blotting results for KANK1 expression in MCF7 and SKBR3 breast cancer cell lines using rabbit polyclonal antibody against human KANK1 (details). Green bands represent KANK1. Morphological characteristics of KANK1 immunohistochemistry in full-face breast cancer tissue. **b**–**d** Normal mammary gland cells showed uniformly strong KANK1 staining. **b** The reactivity of myoepithelial cells (**c**) was lower than those of epithelial cells (white arrow: normal epithelial cells). Invasive cancer cells (**d**) showed uniformly weak KANK1 staining. The reactivity was mainly observed in the cytoplasm. KANK1 protein expression in breast cancer TMA cores (**e, f**). Showing weak staining (**e**, **f**) strong staining in the cytoplasm of cancer cells

KANK1 antibody (rabbit polyclonal SAB500862; SIGMA Company, USA) specificity was assessed by western blot using human BC cell lysates from MCF7 and SKBR3 (obtained from the American Type culture Collection; Rockville, MD, USA). KANK1 antibody (1:500 dilution) was incubated overnight and showed a single band at the expected molecular weight – 90 kDa and mouse β-actin (A5441, Sigma-Aldrich; Clone AC-15; Sigma, UK) at 1:5000 was used as a house-keeping protein (Fig. 1a).

To evaluate the pattern and distribution of KANK1 protein expression, full-face tissue sections (*n* = 14), representative of different BC molecular subtypes and tumour grades, were stained. Tumour samples were arrayed onto TMAs as previously described using the TMA Grand Master® (3D HISTECH®, Budapest, Hungary) [21]. The Novolink Max Polymer Detection system (Leica, Newcastle, UK) was used to detect the immunoreactivity of KANK1. Heat-induced retrieval of antigen epitopes was performed in citrate solution (pH 6.0). KANK1 antibody was incubated at room temperature for 1 h (dilution 1:1500).

### Scoring of KANK1 protein expression

The stained slides were scanned into high-resolution digital images at ×20 magnification using a Nanozoomer scanner (Hamamatsu Photonics, Welwyn Garden City, UK). KANK1 cytoplasmic immunoreactivity was evaluated using the modified H-score taking the staining intensity and percentage of positivity into account. Staining intensity (0–3) was multiplied by the proportion of tumour cells (0–100) stained with each intensity and final scores were obtained, giving a range of 0–300 [22]. Double scoring was assessed blindly by two researchers to evaluate the inter-observer concordance. Intraclass correlation coefficient (ICC) concordance between both observers was 0.9.

### Statistical analysis

SPSS (IBM SPSS Statistic, Version 24.0) was used in statistical analysis. Pearson correlation test was used to evaluate the relationship of *KANK1* mRNA expression with the expression of a set of genes known to be associated with EMT and cancer cell migration (*CDH1, CDH2, TGFB, TWIST2, TWIST1, ZEB2, ZEB1 SLUG, SNAIL, NFKB1, LLGL2, GSK3B, CRUMBS and CTNNB1).* The correlation between KANK1 expression and clinicopathological factors was analysed using Chi-square test. Kaplan–Meier survival curves using the log-rank test were used to assess the prognostic significance of KANK1 expression. Cox proportional hazard method was employed for the multivariate survival analysis. KANK1 mRNA/protein expression did not follow a normal distribution and was dichotomised using median cut-off values (95). The *p* value < 0.05 (two-tailed) was considered statistically significant for clinicopathological parameters and survival. This work was preformed according to REMARK guidelines or tumour prognostic study [23], and approved ethically approval by the North West–Greater Manchester Central Research Ethics Committee under the title: Nottingham Health Science Biobank (NHSB), reference number 15/NW/0685.

## Results

### Clinicopathological significance of KANK1 mRNA expression

**Fig. 2:**
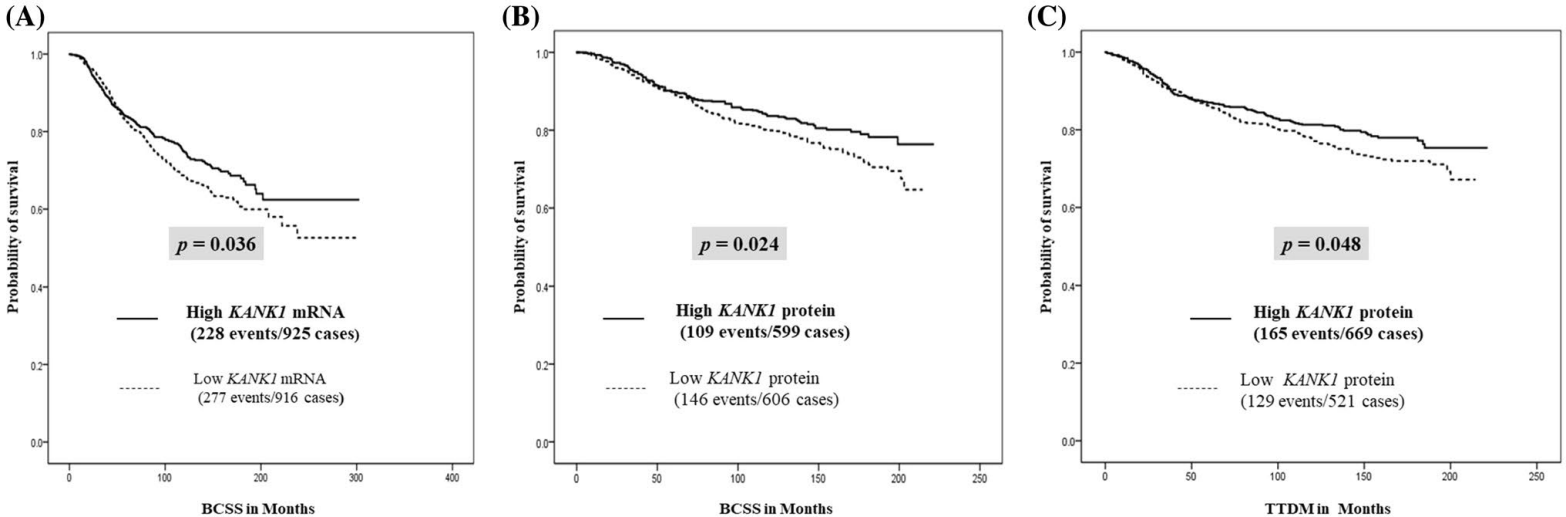
KANK1 patient overall survival and time to distant metastasis. **a** METABRIC cohort, BC overall survival was significantly better in high *KANK1* mRNA expression group than in the low *KANK1* expression group. **b** KANK1 protein expression BC overall survival was significantly better in the high KANK1 protein expression group than in the low expression group. **c** KANK1 protein expression BC TTDM was significantly better in the high KANK1 protein expression group than the low expression group

High *KANK1* mRNA expression was significantly indicative of good prognosis as cases with high *KANK1* mRNA expression had better BCSS outcome compared to low *KANK1* mRNA expression (*p* = 0.036; Fig. 2a). Similar associations were observed in the bc-GenExMiner v4.0 and KM plotter BC datasets (Supplementary Fig. 1a, b). High *KANK1* mRNA expression was also associated with improved outcome when restricting the analysis to subgroups including ER negativity (METABRIC: *p* = 0.007; TCGA: *p* < 0.001), HER2 negativity (METABRIC: *p* < 0.001; TCGA: *p* < 0.001) and LVI negativity (METABRIC: *p* = 0.005; TCGA: *p* = 0.003; Table 1).

**Table 1:** Association of KANK1 mRNA expression with clinicopathological characteristics in the METABRIC (*n* = 1980) and TCGA (*n* = 895) datasets

Parameters	METABRIC cohort			TCGA cohort		
	Low *KANK1*	High *KANK1*	*p* value	Low *KANK1*	High *KANK1*	*p* value
	*N* (%)	*N* (%)		*N* (%)	*N* (%)	
Tumour size						
≤ 2.0 cm	407 (47)	452 (53)	0.033	112 (47)	127 (53)	0.29
>2.0 cm	575 (52)	526 (48)		315 (51)	300 (49)	
Nodal stage						
Negative	502 (49)	533 (519)	0.14	193 (45)	233 (55)	0.007
Positive	487 (52)	451 (48)		231 (55)	192 (45)	
Lymphovascular invasion						
Negative	437 (47)	493 (53)	0.005	258 (46)	301 (54)	0.002
Positive	344 (54)	291 (45)		169 (57)	126 (43)	
Histological grade						
Grade 1 and 2	433 (46)	507 (54)	0.001	229 (49)	235 (51)	0.62
Grade 3	513 (54)	439 (46)		180 (51)	172 (49)	
Oestrogen receptor						
Negative	211 (44)	263 (56)	0.007	66 (35)	119 (65)	< 0.001
Positive	779 (52)	727 (48)		345 (54)	294 (46)	
Progesterone receptor						
Negative	482 (51)	458 (49)	0.3	119 (44)	153 (56)	0.018
Positive	508 (49)	532 (51)		288 (53)	258 (47)	
Human epidermal growth factor receptor 2						
Negative	817 (47)	916 (53)	< 0.001	254 (45)	313 (55)	< 0.001
Positive	173 (70)	74 (30)		94 (71)	39 (29)	

*METABRIC* the molecular taxonomy of breast cancer international consortium, *TCGA* the cancer genome atlas

*KANK1* mRNA overexpression was associated with higher expression of *CDH1 (*METABRIC: *p* = 0.022; TCGA: *p* < 0.001), *CTNNB1* (METABRIC: *p* < 0.001; TCGA: *p* < 0.001); however, *KANK1* mRNA overexpression was correlated with lower LLGL2 (METABRIC: *p* = 0.002; TCGA: *p* < 0.001) (Table 2).

**Table 2:** Correlation of KANK1 mRNA expression with mRNA expression of EMT-related genes

Gene names	METABRIC cohort		TCGA cohort	
	Correlation value	*p* value	Correlation value	*p* value
*CDH1*	0.052	0.022	0.147	< 0.001
*CDH2*	− 0.074	0.001	− 0.035	0.31
*TGFB1*	− 0.043	0.054	− 0.157	< 0.001
*TWIST2*	0.146	< 0.001	− 0.004	0.9
*TWIST1*	0.024	0.29	− 0.44	0.2
*ZEB2*	0.146	< 0.001	0.061	0.075
*ZEB1*	0.008	0.73	0.016	0.65
*SLUG*	0.193	< 0.001	0.143	< 0.001
*SNAIL*	0.014	0.54	0.061	0.075
*NFKB1*	0.069	0.002	0.057	0.095
*LLGL2*	− 0.237	0.002	− 0.118	< 0.001
*GSK3B*	− 0.193	< 0.001	0.094	0.006
*CRUMBS*	− 0.092	< 0.001	61	0.075
*CTNNB1*	0.206	< 0.001	0.222	< 0.001

*METABRIC* the molecular taxonomy of breast cancer international consortium, *TCGA* the cancer genome atlas

### KANK1 protein expression

BC full-face sections showed homogenous cytoplasmic expression of KANK1. KANK1 expression in normal glandular epithelium was uniformly strong (Fig. 1b). KANK1 immunoreactivity of myoepithelial cells was lower than those of glandular epithelial cells (Fig. 1c). In contrast, invasive cancer cells exhibited weaker expression of KANK1 compared to the normal mammary epithelial cells present in some TMA cores (Fig. 1d).

Using the median H-score (95) as a cut-off point, high KANK1 expression was observed in 599/1500 (40%) of tumours (Fig. 1e, f). High KANK1 protein expression was associated with smaller tumour size (*p* = 0.012) and HER2 positivity (*p* = 0.007; Table 3).

**Table 3:** Statistical association between KANK1 protein expression and clinicopathological characteristics of the studies cohort

Parameters	KANK1 protein expression		
	Low	High	*p* value
	*N* (%)	*N* (%)	
Tumour size			
≤ 2.0 cm	382 (47.9)	415 (52.1)	0.012
> 2.0 cm	290 (55.0)	237 (45.0)	
Nodal stage			
Negative	408 (50.0)	408 (50.0)	0.46
Positive	263 (52.1)	242 (47.9)	
Lymphovascular invasion			
Negative	461 (49.5)	471 (50.5)	0.15
Positive	211 (53.8)	181 (46.2)	
Histological grade			
Grade 1 and 2	360 (51.6)	337 (48.4)	0.49
Grade 3	312 (49.8)	315 (50.2)	
Nottingham Prognostic Index			
Good prognostic group	198 (47.4)	220 (52.6)	0.22
Moderate prognostic group	361 (52.8)	323 (47.2)	
Poor prognostic group	112 (51.4)	106 (48.6)	
Oestrogen receptor			
Negative	133 (47.3)	148 (52.7)	0.2
Positive	540 (51.7)	505 (48.3)	
Progesterone receptor			
Negative	276 (49.6)	281 (50.4)	0.47
Positive	393 (51.6)	369 (48.4)	
Human epidermal growth factor receptor 2			
Negative	599 (52.6)	539 (47.4)	0.00074
Positive	74 (39.4)	114 (60.6)	
Triple negative breast cancer			
Negative	571 (51)	549 (49)	
Positive	102 (49)	104 (51)	0.699

Those patients with tumours showing high KANK1 protein expression had significantly better 10 years BCSS (*p* = 0.024; Fig. 2b) and longer TTDM (*p* = 0.048; Fig. 2c) compared with those patients showing low/reduced KANK1 expression. Multivariate analyses indicated that high KANK1 expression is correlated (< 0.05) with better outcome in terms of longer BCSS and TTDM, independent of other established prognostic variables including tumour size, Nottingham grade, nodal stage, LVI, ER status, PR status and HER2 status (Table 4).

**Table 4:** Multivariate Cox proportional hazard regression analysis for predictors of BCSS and time to distant metastasis (TTDM) in the Nottingham BC cohort

Factors	BCSS			TDDM		
	Hazard Ratio	95% CI	*p* value (< 0.05)	Hazard ratio	95% CI	*p* value (< 0.05)
KANK1 expression	1.4	1.072–1.786	**0.0013**	1.3	1.02–1.64	**0.033**
Tumour size	0.78	0.59–1.02	0.065	0.68	0.53–0.88	**0.003**
Tumour grade	1.62	1.26–2.08	**<** **0.0001**	1.35	1.08–1.68	**0.008**
Tumour stage	1.78	1.50–2.13	**<** **0.0001**	1.65	1.40–2.00	**<** **0.0001**
Lymophvascular invasion	0.54	0.41–0.71	**<** **0.0001**	0.55	0.43–0.88	**<** **0.0001**
ER status	1.11	0.79–1.57	0.544	0.99	0.71–1.38	0.95
PR status	0.57	0.42–0.78	**<** **0.0001**	0.68	0.50–0.88	**0.004**
HER2 expression	0.76	0.56–1.04	0.09	0.67	0.50–0.90	**0.007**

**Fig. 3:**
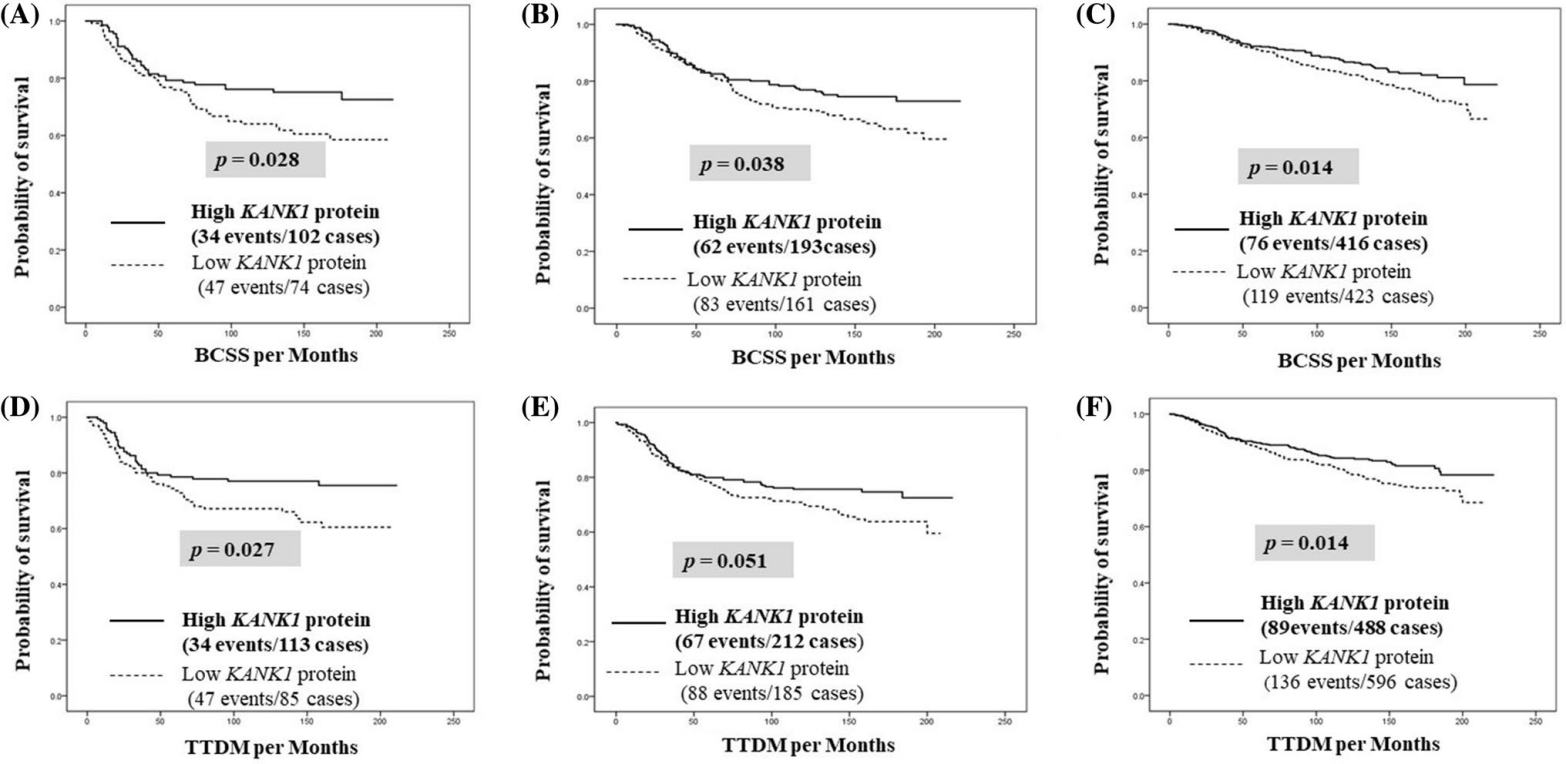
Molecular BC subtypes overall survival and time to distant metastasis. **a** ER-negative BC patients’ overall survival. **b** PR negativity BC patients’ overall survival. **c** HER2-negative BC patients’ overall survival. **d** ER-negative BC TTDM patients. **e** PR-negative BC TTDM patients. **f** Her2-negative BC TTDM patients

**Fig. 4:**
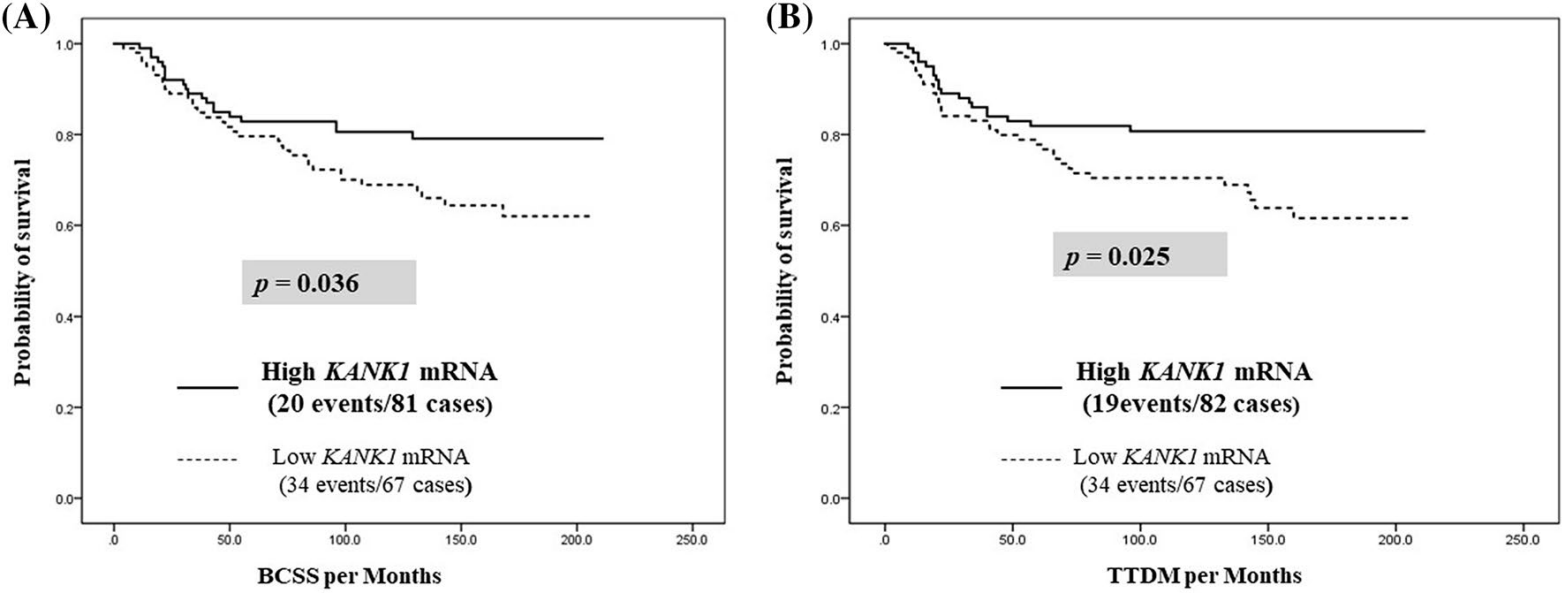
Patients’ outcomes of Triple Negative BC subtype. **a** BCSS survival and **b** time to distant metastasis

When we stratified our BC cohort based on hormonal receptor and HER2 expression, overexpression of KANK1 protein was predictive of longer BCSS in the receptor-negative subgroups (*p* = 0.024, *p* = 0.038 and *p* = 0.014 for ER−, PR− and HER2− tumours, respectively; Fig. 3a–c). TTDM showed similar association in both ER and HER2-negative BC (*p *= 0.027 and *p *= 0.014) (Fig. 3d, e). Importantly, when exploring the value of KANK1 protein expression in TNBC (*n* = 203), high KANK1 expression was also associated with prolonged survival (BCSS: *p* = 0.036 and TTDM *p* = 0.025; Fig. 4).

## Discussion

This study has robustly demonstrated that high KANK1 expression is associated with good prognostic characteristics and improved BC patients’ outcomes, which is in agreement with other cancers including gastric [11], nerve [24] and lung [25]. Our study also showed that high *KANK1* mRNA expression showed improved survival time in the aggressive and clinically relevant subgroups of BC, namely ER, PR and HER2-negative tumours. It was also strongly associated with clinicopathological variables characteristic of good prognosis including LVI negativity and lower grade, highlighting a potential tumour suppressive role in BC.

In the current study, high *KANK1* mRNA expression was associated with ER, PR and HER2 negativity. This is consistent with KANK1 protein, except for HER2. This discrepancy in KANK1:HER2 expression between the protein and transcript levels may be attributable to the nature of the cohort, complicated post-transcriptional mechanisms and proteins may differ substantially in their in vivo half-lives [24, 25]. However, due to the relatively small sample size of the HER2-positive subgroup, further confirmation in larger cohorts of both HER2-positive and HER2-negative cases is required to determine the exact role of KANK1 in HER2-positive BC.

Nonetheless, when investigating the role of *KANK1* mRNA expression with well-established EMT transcription factors, our data showed a negative correlation between *KANK1* mRNA expression and other EMT genes (*TGFB1, CDH2, LLGL2* and *CTNNB1*). On the other hand, high *KANK1* mRNA expression showed a significant positive association with E-cadherin gene (*CDH1)*, and these findings suggest that high *KANK*1 expression is involved in reducing tumour cell migration and influencing the LVI process through reducing the RhoA/ROCK pathway, which has a well-known role in controlling cancer cell migration [7]. *TGFB1* acts as an oncogene in tumour progression by inducing cell invasion, dissemination to distant sites and augmenting angiogenesis. *CDH2* and *LLGL2*, which play an important role in EMT activation, were negatively correlated with *KANK1* high expression. This suggested that the EMT activation is prohibited by the presence of *TGFB1*, *CDH2* and *LLGL2*. Chen et al. showed in gastric cancer, increased KANK1 expression was associated with smaller tumour size; results in agreement with our study results in both mRNA and protein levels, implying its role in decreasing cellular proliferation. Similarly, KANK1 may regulate the cell proliferation through inhibiting the phosphorylation of PI3 K/AKT proteins [26]. Smaller tumour size and negative association with *TGFB1, CDH2* and *LLGL2* strengthen the tumour suppressive role of KANK1.

In the whole BC cohort, high KANK1 protein expression was an independent prognostic marker for improved patients’ outcomes in terms of both BCSS and TTDM. Among subgroups, high expression of KANK1 protein appears to play the most significant survival role in TNBC. As TNBCs are highly resistance to chemotherapy compared to other BC types and strongly associated with worse clinical outcome, our results may indicate the promising role of KANK1 in this aggressive subtype regarding benefit from neoadjuvant chemotherapy and improved overall survival [27].

Our results suggest that loss of expression of KANK1 promotes BC progression. This is in concordance with previous reports indicating that reduced expression of KANK1 facilitates metastasis in different types of cancer and further reinforces its role as a prognostic indicator [11, 24, 25].

In summary, high KANK1 expression in BC is associated with favourable prognostic parameters and is an independent prognostic factor with prolonged patient survival. KANK1 appears to play a role in inhibiting tumour cells proliferation, migration, invasion and metastasis. Further functional studies to decipher the role of KANK1 and its mechanism of action as a tumour suppressive driver of invasive BC is warranted.

## Electronic supplementary material

Below is the link to the electronic supplementary material.

Supplementary material 1 (JPEG 70 kb)

Supplementary material 2 (DOCX 21 kb)

## Data Availability

The authors confirm the data that have been used in this work are available on reasonable request.
