# Supplementary figures and images for: Prognostic significance of KN motif and ankyrin repeat domains 1 (KANK1) in invasive breast cancer
Source: Breast Cancer Res Treat. 2019 Nov 2;179(2):349–57. doi: 10.1007/s10549-019-05466-8 (PMC6987050; doi:10.1007/s10549-019-05466-8)

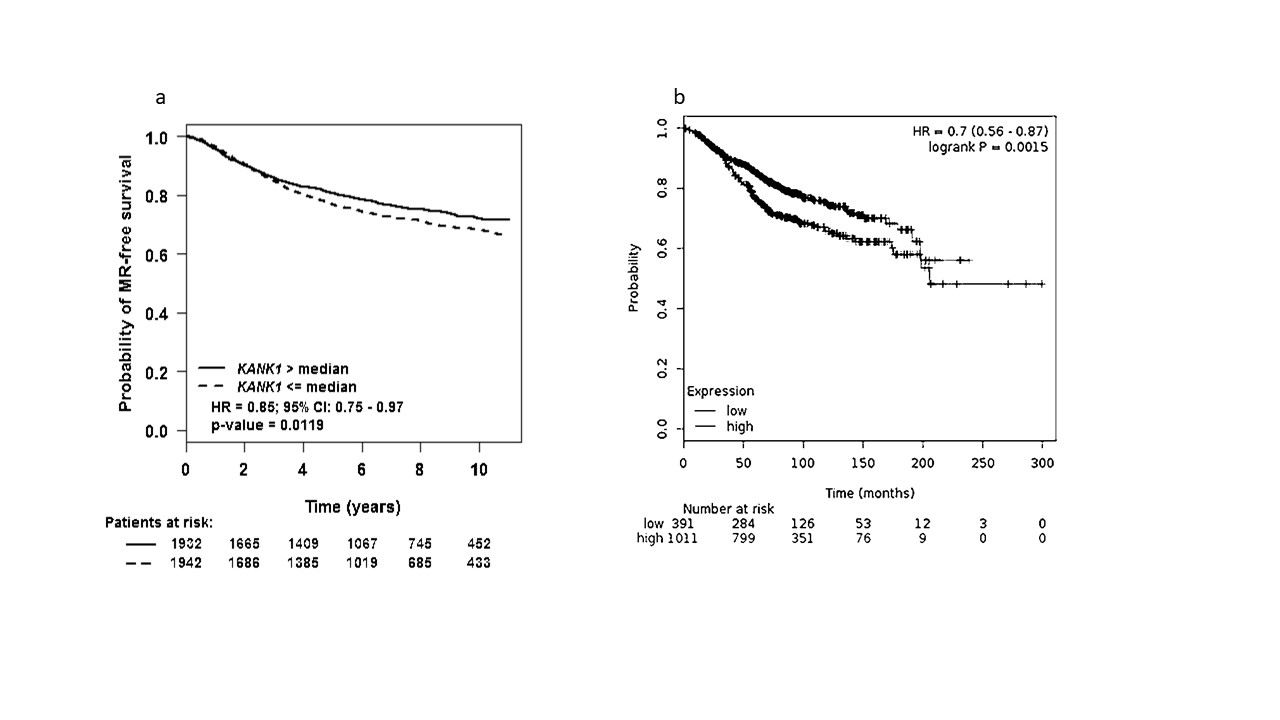

Supplement: Supplementary file 1 — Supplementary material 1 (JPEG 70 kb) [file 10549_2019_5466_MOESM1_ESM.jpg]
