# Supplementary material for: Prognostic significance of KN motif and ankyrin repeat domains 1 (KANK1) in invasive breast cancer
Source: Breast Cancer Res Treat. 2019 Nov 2;179(2):349–57. doi: 10.1007/s10549-019-05466-8 (PMC6987050; doi:10.1007/s10549-019-05466-8)
Supplement: Supplementary file 2 — Supplementary material 2 (DOCX 21 kb) [file 10549_2019_5466_MOESM2_ESM.docx]

**Supplementary Table 1.** Clinicopathological parameters of the METABRIC and Nottingham validation series.

| **Pathological parameters** | **METABRIC series**  **N (%)** | **Nottingham set**  **N (%)** |
| --- | --- | --- |
| **Age** | | |
| ˂ 50 years | 424 (21.4) | 469 (30.5) |
| ≥ 50 years | 1426 (78.6) | 1070 (69.5) |
| **Tumour size** | | |
| < 2cm | 623(31.8) | 939 (61.1) |
| ≥ 2cm | 1337(68.2) | 599 (38.9) |
| **Grade** | | |
| 1 | 169 (9.0) | 231 (15.0) |
| 2 | 770 (40.7) | 622 (40.4) |
| 3 | 952 (50.3) | 685 (44.5) |
| **Tumour types** | | |
| Ductal (including mixed) | 1545 (83.6) | 1335 (86.9) |
| Lobular | 148 (8.0) | 120 (7.8) |
| Medullary-like | 32 (1.7) | 13 (0.8) |
| Miscellaneous | 12 (0.6) | 9 (0.6) |
| Special type | 113 (6.1) | 60 (3.9) |
| **Vascular invasion** | | |
| Definite | Not available | 451 (29.3) |
| Negative/Probable |  | 1086 (70.7) |
| **Lymph Node Stage** | | |
| 1 | 1035 (52.5) | 955 (62.2) |
| 2 | 623 (31.5) | 428 (27.9) |
| 3 | 315 (16.0) | 153 (10.0) |
| **ER** | | |
| Negative | 472 (23.8) | 300 (19.5) |
| Positive | 1508 (76.2) | 1240 (80.5) |
| **PgR** | | |
| Negative | 938 (47.4) | 612 (41.8) |
| Positive | 1042 (52.6) | 853 (58.2) |
| **HER2** | | |
| Negative | 1734 (87.5) | 1376 (89.9) |
| Positive | 246 (12.5) | 155 (10.1) |
